# A Review: Pharmacological Effect of Natural Compounds in *Diospyros kaki* Leaves from the Perspective of Oxidative Stress

**DOI:** 10.3390/molecules29010215

**Published:** 2023-12-30

**Authors:** Chong Hong, Xu Wang, Jianjian Xu, Jianxing Guo, Houlin Peng, Yan Zhang

**Affiliations:** 1 Key Laboratory of Computational Chemistry-Based Natural Antitumor Drug Research & Development, School of Traditional Chinese Materia Medica, Shenyang Pharmaceutical University, Shenyang 110016, China; 18720397992@163.com (C.H.); xujianj2023@126.com (J.X.); 15504058325@163.com (J.G.); 15041466766@163.com (H.P.); 2 Department of Breast Surgery, The First Affiliated Hospital of China Medical University, Shenyang 110001, China; soloman2003@163.com; 3 Lonch Group Wanrong Pharmaceutical Co., Ltd., Yuncheng 100176, China

**Keywords:** leaves of *Diospyros kaki*, natural products, oxidative stress

## Abstract

Oxidative stress is caused by an imbalance between reactive oxygen species and antioxidant levels. Current research suggests that oxidative stress is one of the key factors in the development of many chronic diseases, and it has been a concern for many years. Many natural compounds have been studied for their special free-radical-scavenging properties. The major chemical constituents of the leaves of *Diospyros kaki* are flavonoids and triterpenoids, both of which are potential antioxidants that can prevent damage caused by reactive oxygen species or reactive nitrogen species and ameliorate diseases associated with oxidative stress. In addition to the major constituents such as flavonoids and triterpenoids, the leaves of *Diospyros kaki* include compounds such as phenylpropanoids, alkaloids, phenolic acids, and terpenes. Studies have shown these compounds have certain antioxidant and neuroprotective activities. Experiments have shown that flavonoids or the extracts from the leaves of *Diospyros kaki* have a variety of good pharmacological activities, which could activate oxidative stress and mitochondrial apoptosis, inhibit the proliferation of human prostate cancer cells and induce apoptosis. It also could achieve the effect of anti-cancer cell proliferation and induce apoptosis by regulating oxidative stress. The main chemical substance of the leaves of *Diospyros kaki* regulating oxidative stress may be these multi-hydroxyl structure compounds. These natural products exhibit significant antioxidant activity and are an important basis for the leaves of *Diospyros kaki* to treat human diseases by regulating oxidative stress. This review summarizes the structural types of natural products in the leaves of *Diospyros kaki* and elaborates the mechanism of the leaves of *Diospyros kaki* in neuroprotection, anti-diabetes, renal protection, retinal degenerative diseases, and anti-cancer from a new perspective of oxidative stress, including how it supplements other pharmacological effects. The chemical constituents and pharmacological effects of the leaves of *Diospyros kaki* are summarized in this paper. The relationship between the chemical components in the leaves of *Diospyros kaki* and their pharmacological effects is summarized from the perspective of oxidative stress. This review provides a reference for the study of natural anti-oxidative stress drugs.

## 1. Introduction

Oxidative stress has attracted a lot of attention since it was proposed in 1985. Oxidative stress refers to a state in which there is an imbalance between oxidation and antioxidant activity in the body, with a tendency toward oxidation. Oxygen radicals are an unavoidable byproduct of many biochemical processes, which are intentionally formed in some cases. As in activated neutrophils, they are produced in the body by environmental electromagnetic radiation and directly as oxidizing pollutants such as ozone and nitrogen dioxide [1]. Oxidative stress causes excessive oxygen free radicals to attack biomolecules such as lipids, proteins, and DNA, which can lead to tissue damage [2]. Oxidative stress has been implicated in the pathogenesis of a variety of common diseases, including stroke, hypertension, diabetes, neurodegenerative diseases, and malignancies [3,4,5]. In the study of a variety of diseases, many natural products have been found to be effective in regulating oxidative stress and thus exert anti-cancer properties [6] and the ability to treat intracerebral hemorrhage [7], diabetes, and neurodegenerative diseases due to an excessive inflammatory response [8]. This may be due to the unique free-radical-scavenging effect of plant-derived natural products [9]. Known studies have shown that medicinal plants are an important source of antioxidants that can help fight oxidative stress and modulate various pharmacological processes, including oxidative stress and inflammation [10].

The persimmon (*Diospyros kaki* L.) is a plant of the genus *Diospyros* Linn. in the family Ebenaceae, native to the Yangtze River basin in China. Persimmon leaves are dry or fresh leaves of persimmon trees [11]. As a natural product beneficial to human health, persimmon leaves have always played a key role in the long history of human health development. In China, persimmon leaves have long been used as traditional Chinese medicine. Their application was first recorded in the Diannan Bencao of the Ming Dynasty: Treatment of Eczema with frost and leaves [12]. Persimmon leaves were commonly used to treat cough, hemorrhage, hypertension, stroke, and other diseases [13], and persimmon leaf tea was drunk as a natural dietary supplement in Japan, South Korea, China, and other Asian countries [14]. Persimmon leaves are used in many medical and health-related products, such as cosmetics and the clinical medicine Naoxinqing [15,16]. However, as a natural product with abundant resources, persimmon leaves still have unlimited potential for the healthy development of human beings.

Persimmon has a high research value because it contains rich and diverse compounds and other nutrients [17,18]. Persimmon leaves are reported to be rich in flavonoids, terpenes, lignin, coumarins, alkaloids, polysaccharides, and volatile oils [19] but also contain many nutrients such as vitamin C, choline, several amino acids, calcium, iron, and zinc [20]. The abundance of phenolics in persimmon leaf extract gives it excellent antioxidant activity [21,22], which can also be inferred from the antioxidant activity of other natural products related to persimmon leaves (persimmon peel, persimmon) [23,24]. With the development of modern pharmacological research on persimmon leaves, it is much clearer that persimmon leaves have a wide range of pharmacological effects, including anti-cancer, anti-inflammatory, anti-allergic, hypoglycemic, antihypertensive, neuroprotective, cardiovascular protective, etc. [25]. Many of these pharmacological activities depend on the high antioxidant activity of persimmon leaf extracts. The intake of antioxidants from natural products has extraordinary benefits for human health [25,26].

These natural products from persimmon leaves may help regulate oxidative stress, opening up new possibilities for the treatment of a variety of diseases. Therefore, it is necessary to elaborate and summarize the phytochemistry and pharmacological activity of persimmon leaves, the pharmacological activities of persimmon leaf extracts that work through the regulation of oxidative stress mechanisms, and other biological activities and their mechanisms. In this paper, from the perspective of the structure types of natural products and their pharmacological activities, the types of chemical components in persimmon plants are comprehensively introduced, the particularity of the compound structure is discussed, and the pharmacological activities of persimmon leaves in oxidative stress are emphatically discussed. This paper fully summarizes the pharmacological activities of the chemical components of persimmon leaves, updates the knowledge status in this field, establishes a scientific framework, and provides some new ideas for the study of oxidative stress.

## 2. Phytochemistry of Persimmon Leaves

### 2.1. Plant Characteristics and Spread

Persimmons are deciduous or evergreen trees or shrubs with greyish-green or yellowish-brown oval or obovate leaves [27]. Persimmon accessions are very rich, with 450 species, which are widely distributed in Asian countries such as China, Japan, and Korea [28]. There are 57 species of persimmon in China, which are mainly distributed from Southwest to Southeast China, with a cultivation history of more than 3000 years [29]. Persimmon is an economic crop with huge product value and has wide commercial and medical values for derivative products such as persimmon leaf, persimmon stem, persimmon fruit, and persimmon cream [30].

### 2.2. Chemical Composition of Persimmon Leaves

**Figure 1:**
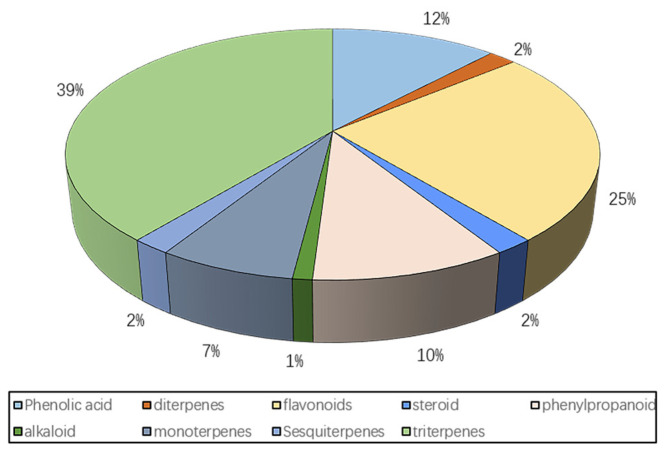
Chemical composition of persimmon leaves.

This article summarizes the structures of all flavonoids, terpenes, phenylpropanoids, steroids, alkaloids, and phenolic acids that have been isolated from persimmon leaves. In addition, there are many nutrients found in persimmon leaves, such as fatty acids, polysaccharides, cellulose, etc., which are only briefly discussed here (Figure 1).

#### 2.2.1. Flavonoids

**Figure 2:**
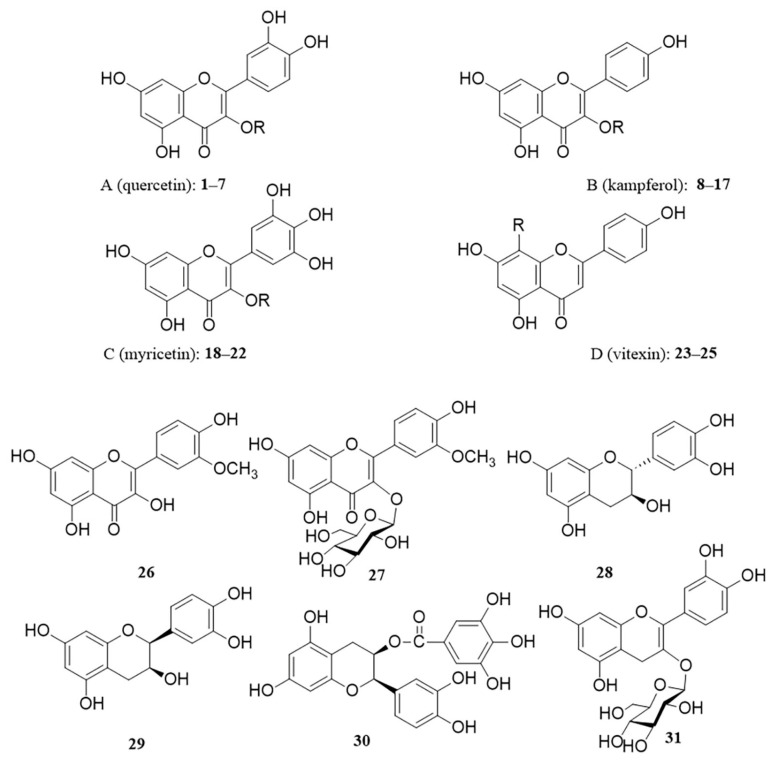
Structure of flavonoids in persimmon leaves.

Flavonoids are the main chemical components of persimmon leaves and important active substances. Flavonoids from persimmon leaves possess tyrosinase-inhibitory activity [31]. Persimmon leaf flavonoids (myricetin and its glycosides) inhibit the formation of *N*-nitrosamines and remove nitrite from the human body [32,33]. These studies have shown that flavonoids in persimmon leaves are important active substances. Flavonoids are one of the major components in persimmon leaves. Sun Huapeng measured the average total flavonoid content of dried persimmon leaves of 15 varieties as 59.77 mL/g [34]. Judging from the process of separating and obtaining flavonoids, persimmon leaves are almost extracted with ethanol, then extracted with ethyl acetate, and flavonoid monomer compounds are obtained by modern separation and enrichment methods. There are 31 flavonoids that have been isolated from persimmon leaves. The flavonoids in persimmon leaves are mainly glycosides or aglycones of quercetin (A), kaempferol (B), myricetin (C), and vitexin (D). Moreover, some flavonoids other than the above four classes were also present (Figure 2, Table 1). Interestingly, the antioxidant activity of galloyl-substituted flavonol glycosides was found to be much higher than that of non-galloyl-substituted flavonoid glycosides [35].

#### 2.2.2. Triterpenes

**Figure 3:**
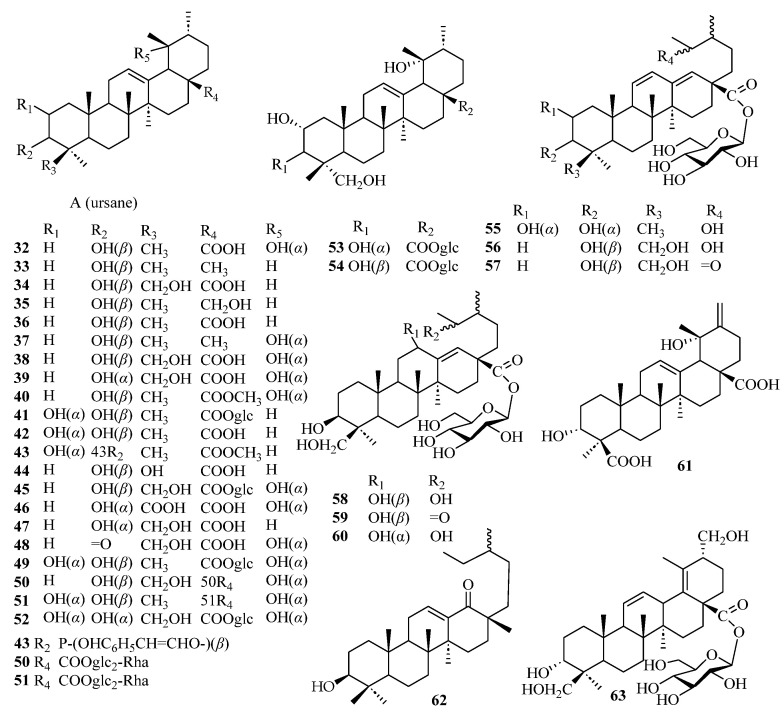
Structures of ursane-type triterpenes.

**Figure 4:**
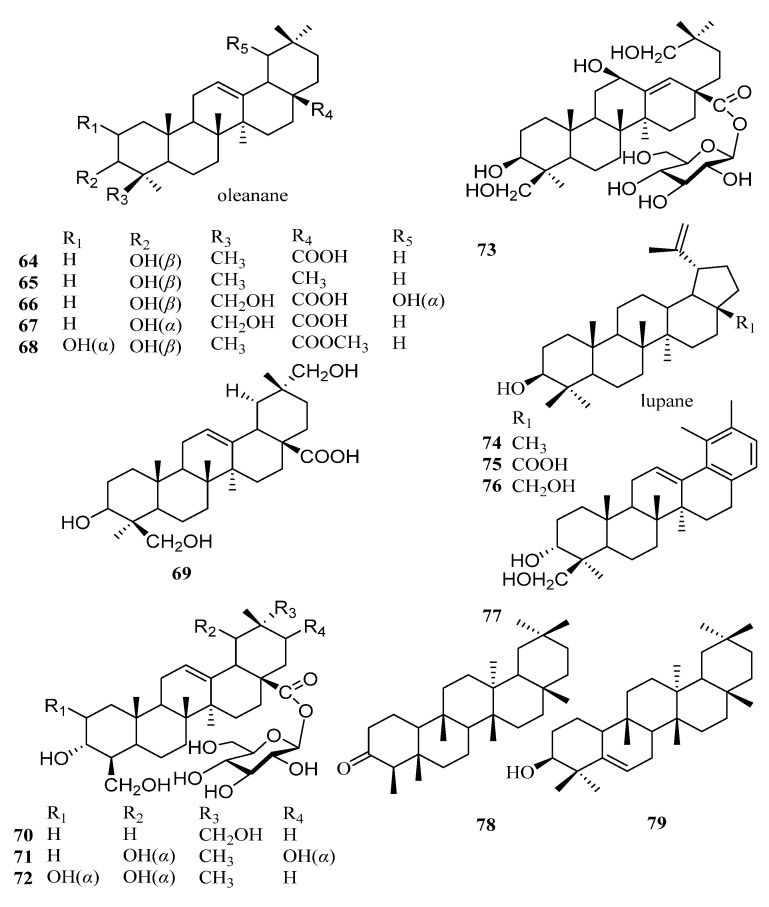
Structures of other types of triterpenoids in persimmon leaves.

Triterpenes are structurally diverse organic compounds characterized by polycyclic skeletons modified in various ways. Saponins are triterpenes combined with natural sugars. These natural products are of great importance in chronic diseases associated with oxidative stress, such as diabetes and neurodegenerative diseases, as well as in anti-inflammatory, hepatoprotective, antibacterial, antiviral, immunosuppressive, and other aspects [43,44]. It is worth noting that most of the triterpenoids in persimmon leaves are distributed in the ethyl acetate extraction layer of the ethanol extract. At present, 32 ursane-type triterpenes have been isolated from persimmon leaves—21 compounds with feature A and 11 other ursane-type triterpenes. It is interesting to note that many triterpenes isolated from persimmon leaves have the structure of E-ring cracking at positions 18 and 19, which are different from common triterpene skeletons (Figure 3, Table 2). These 18 and 19 secoursane triterpenoids are also characteristic compounds in persimmon leaves. There are other types of triterpenes in persimmon leaves, including oleanane and lupinane (Figure 4, Table 3).

#### 2.2.3. Other Natural Products in Persimmon Leaves

**Figure 5:**
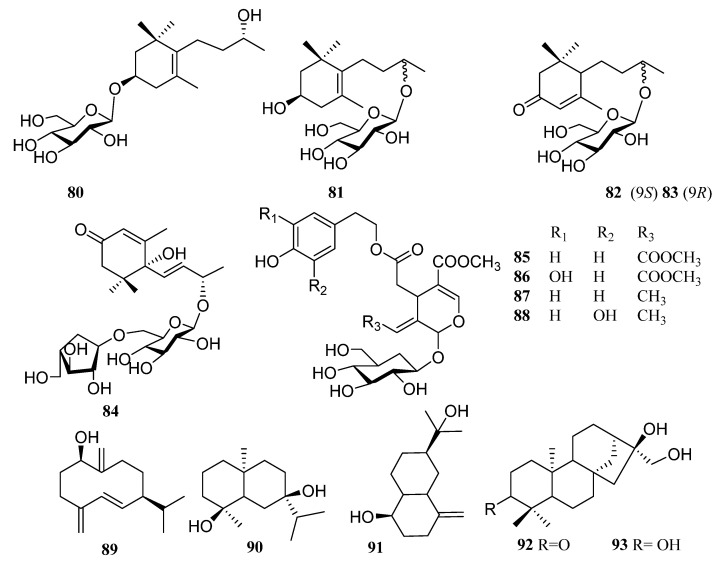
Structures of monoterpenes, sesquiterpenes, and diterpenes in persimmon leaves.

**Figure 6:**
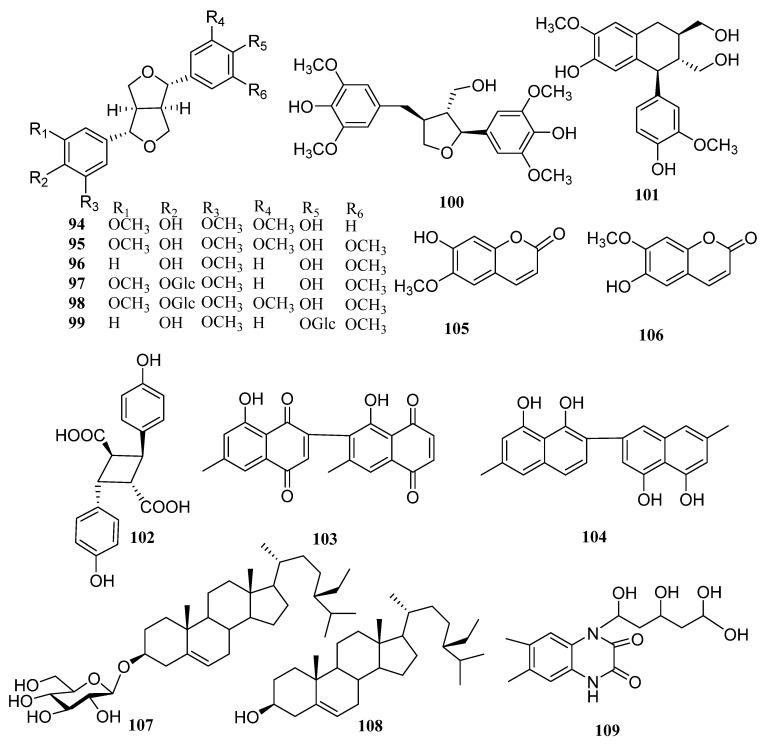
Structures of phenylpropanoids, steroids and alkaloids in persimmon leaves.

**Figure 7:**
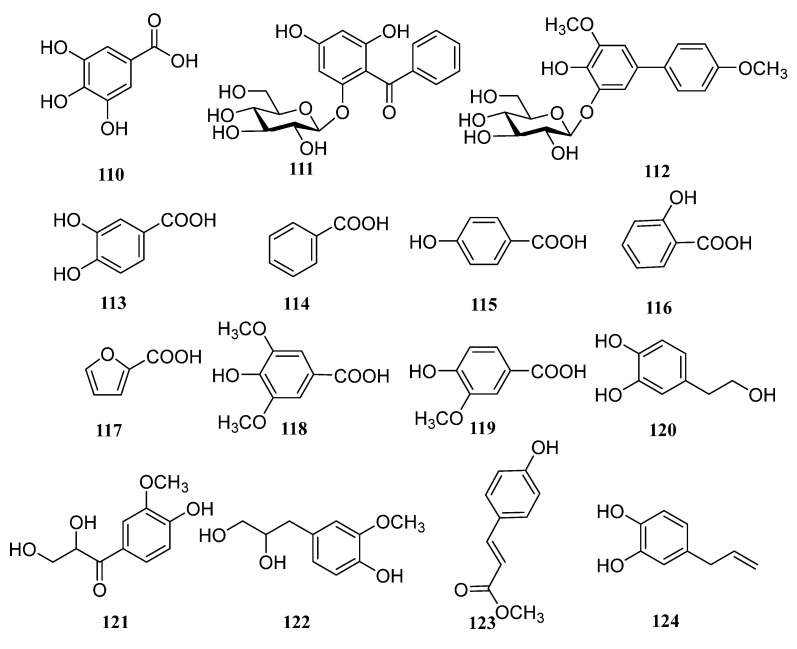
Phenolic acid structures in persimmon leaves.

In addition to the above triterpenoid structure, there are also some monoterpenes, sesquiterpenes and diterpenoids in persimmon leaves (Figure 5, Table 4). Phenylpropanoids (**94**–**106**), steroids (**107**, **108**), alkaloids (**109**), and a large number of phenolic acids (**110**–**124**) are also found in persimmon leaves (Figure 6, Table 5) (Figure 7, Table 6). These natural products show certain antioxidant and neuroprotective activities, and more phenolic hydroxyl groups will improve their antioxidant capacity [55,56]. In particular, studies have shown that vomifoliol 9-*O*-α-arabinofuranosyl (1→6)-β-d-glucopyranoside (**84**) isolated from persimmon leaves can inhibit α-glucosidase activity and has some therapeutic significance in type 2 diabetes [57]. Polysaccharides in persimmon leaves are also important nutrients that play a significant role in anti-cancer, anti-osteoporosis, and immune regulation [58,59,60].

## 3. Diseases Related to Oxidative Stress

**Figure 8:**
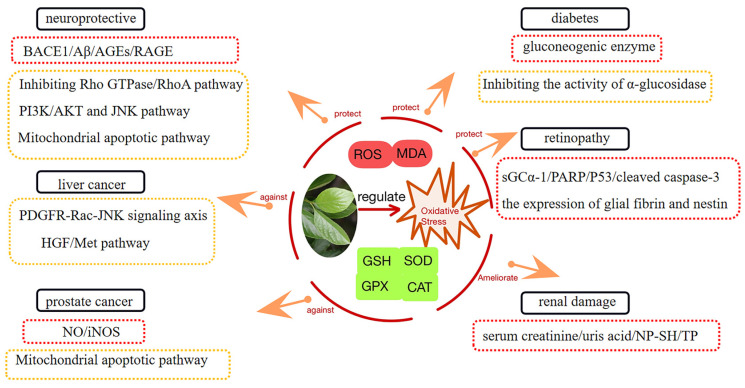
Persimmon leaf is involved in the regulation of oxidative stress to exert therapeutic effects in some diseases.

It is well known that the excessive accumulation of reactive oxygen species (ROS) is detrimental to human health when the ROS are produced in excess and the antioxidant system is unable to correct the imbalance between the ROS [66]. ROS include superoxide anions, hydroxyl radicals, and hydrogen peroxide [67]. ROS production is dominated by mitochondrial oxidative phosphorylation and the nicotinamide adenine dinucleotide phosphate oxidase systems [68]. Mitochondria are the main source of ROS in cells. Mitochondrial reactive oxygen species have been implicated in the pathogenesis of many diseases and are involved in important physiological processes such as cell proliferation, differentiation, aging, and apoptosis [69]. The excessive accumulation of ROS leads to oxidative stress, which has been implicated in the pathogenesis of many diseases, including diabetes, cardiovascular diseases, cancer, and neurodegenerative diseases [70,71,72]. The above diseases related to oxidative stress are also the focus of research on persimmon leaves due to their antioxidant activity (Figure 8).

In the study of the antioxidant activity of the total flavone extract of persimmon leaves (TFPL), it was found that the TFPL was able to significantly reduce the levels of ROS and malondialdehyde in mouse osteoblast mouse embryonic osteoblasts cells, while the activities of catalase (CAT), superoxide dismutase (SOD) and glutathione peroxidase (GSH-Px) were enhanced. The TFPL has better reducing ability and free-radical-scavenging ability and is dose-dependent, even more significantly than rutin [73]. Rats, after gamma irradiation, develop a liver injury and increased levels of oxidative stress and metabolic abnormalities; treatment with persimmon leaf extract (PL) resulted in reduced levels of oxidative stress, indicated not only by decreased malondialdehyde (MDA) levels and xanthine oxidase (XO) activity but also by increased glutathione (GSH) levels and SOD, CAT and xanthine dehydrogenase (XDH) activities. In addition to reducing liver damage, PL (1000mg/kg BW/day) can inhibit glucose concentration, increase insulin levels, improve dyslipidemia, and significantly reduce atherosclerosis indicators compared with the control group (Table 7) [74].

### 3.1. Diabetes and Its Complications

The water extract of persimmon leaf (PLE) was administered to alloxan-induced hyperglycemic rats for two weeks. The experimental group showed a significant hypoglycemic effect; this was reflected in the decrease in fasting blood glucose (*p* < 0.01) and the increase in liver glycogen content (*p* < 0.01) [75]. After the administration of PLE, the serum levels of total cholesterol (TC), triglyceride (TG) and low-density lipoprotein cholesterol (LDL-C) in hyperlipidemia rats decreased, the levels of high-density protein cholesterol (HDL-C) increased, and the activities of SOD, GSH-Px and hepatic lipase (HL) increased significantly, indicating that PLE had a significant effect on lowering blood lipids. This study indicated that the mechanism of blood lipid-lowering by PLE may be related to eliminating oxygen free radicals in the body and improving HL levels [76]. To investigate the underlying mechanisms by which PL ameliorates hyperglycemia, hyperlipidemia, and hepatic steatosis in type 2 diabetes, Un Ju Jung observed that PL ameliorated plasma and hepatic oxidative stress, resulting in reduced hepatic fatty acid oxidation, hyperlipidemia, and hepatic steatosis, after adding PL (5%, *w*/*w*) to the normal diet of C57BL/KsJ-db/db mice for 5 consecutive weeks. Gluconeogenic enzyme activity was suppressed in the liver, while glycogen content and glucokinase activity and its mRNA expression levels were increased, which may shed light on the underlying mechanism of PL against hyperglycemia [77]. PLE is also able to exert hypoglycemic effects by inhibiting α-glucosidase activity, increasing antioxidant capacity, and maintaining β-cell function [78].

Renal damage caused by oxidative stress is often associated with the onset of diabetes [79]. The carbon tetrachloride (CCl_4_)-induced generation of ROS and toxic free radicals causes nephrotoxicity [80]. However, the treatment of Swiss albino rats with CCl_4_-induced nephrotoxicity treated with PLE resulted in a significant decrease in serum creatinine, MDA, and uric acid levels, but this increased total protein (TP) and nonprotein sulfhydryl (NP-SH) levels, showing renoprotective effects. The most effective natural products include kaempferol, quercetin, astragaloside, and rutin [81]. In treating renal oxidative damage in type 2 diabetic mice, Myung-Sook Choi found that persimmon leaf powder could reduce the levels of oxidative stress markers, improve antioxidant enzyme (SOD, CAT, glutathione peroxidase (GPX)) activities and mRNA expression, alleviate oxidative stress, and thereby improve renal protection [82].

Retinopathy is a microvascular complication of diabetes, and oxidative stress is a key factor closely associated with the disease [5]. The retina is one of the tissues with the highest oxygen consumption [83]. The excessive accumulation of reactive oxygen species leads to retinal damage and even blindness, which is also the reason why retinal damage is often caused by oxidative stress [84]. Supplementation with antioxidants from natural products is important in the fight against retinal degeneration [85]. During the treatment of mice with microbead-induced ocular hypertension, the ethanol extract of persimmon leaves showed activity in treating retinal degenerative diseases by upregulating soluble guanylate cyclase (sGCα-1), reducing retinal ganglion cell loss and optic nerve damage [86]. In the mouse model of *N*-Methyl-*N*-nitrosourea-induced retinal degeneration, the retinal thickness of mice increased after treatment with an oral ethanol extract of *Diospyros kaki* (EEDK); based on the antioxidant properties of EEDK, the expression of endogenous antioxidant enzymes (SOD, GPX) was upregulated, and the expression of glial fibrin and nestin in Müller and astrocyte cells was inhibited, showing a protective effect against oxidative stress-induced cell death. It is worth noting that quercetin played an important role in this process [87]. The treatment of retinal ganglion cells (RGC-5) cells with EEDK significantly increased cell viability and inhibited the upregulation of poly (ADP-ribose) polymerase (PARP), and P53 and cleaved caspase-3 proteins were inhibited, reducing oxidative stress and apoptosis. Moreover, EEDK also has a certain protective effect on retinal degeneration caused by mechanical injury [88].

### 3.2. Neuroprotective Activity

Flavonoids from the leaves of *Diospyros kaki* (FLDK-P70) can reduce hypoxia reoxygenation-induced neuronal death and apoptosis in a dose-dependent manner, and the underlying mechanism may be related to the antioxidant activity of the flavone [89]. For H_2_O_2_-induced apoptosis-like injury in mouse neuroblastoma–rat glioma hybrid cells (NG108-15 cells), treatment with FLDK-P70 could improve redox imbalance, reduce MDA and ROS levels, and alleviate the damage of oxidative stress to nerve cells by upregulating the expression of B-cell lymphoma-2 (Bcl-2), a suppressor protein of apoptosis [90]. After the oral administration of FLDK to amyloid precursor protein/presenilin1 (APP/PS1) transgenic mice, Amyloid-β peptide (Aβ) production was reduced, the expression of β-site amyloid precursor protein cleavage enzyme 1 (BACE1) was downregulated, antioxidant enzyme activities were increased, and lipid peroxidation products were decreased. MDA and inflammatory mediators suggest that FLDK ameliorates cognitive deficits in mice by regulating oxidative stress and anti-inflammatory activities, as well as by removing Aβ deposits [91]. In addition, FLDK has a synaptic protective function that may be mediated by regulating the synapse-associated protein Rho guanosine triphosphatase (Rho GTPase), thereby inhibiting the expression of the downstream protein Ras homolog gene family member A (RhoA) and improving synaptic dysfunction and reversing memory impairment [92]. D-galactose-induced oxidative stress and neuroinflammation-mediated brain senescence in mice can be inhibited by FLDK, depending on the ability of FLDK to reduce the level of oxidative stress and inhibit the expression of advanced glycation end products (AGEs) and AGEs receptors (RAGE) and D-galactose-induced neuroinflammation. FLDK also ameliorates synapse-associated protein damage by inhibiting the phosphatidylinositol 3-kinase (PI3K/AkT) and C-Jun *N*-terminal kinase (JNK) apoptotic signaling pathways [92]. In addition to flavonoids, triterpenoids and other compounds in persimmon leaves also show excellent neuroprotective activity [46,55]. Persimmon leaf ethyl acetate extract (EAPL) alleviates the apoptosis of hippocampal neurons by regulating oxidative stress and mitochondrial-mediated apoptosis-associated proteins; this includes a decrease in phospho-C-Jun *N*-terminal kinases and capase-3 expression and a decrease in the relative ratio of Bcl-2-associated X protein. The natural products analyzed for their effect on Alzheimer’s were mainly flavones and triterpenes [93].

### 3.3. Anti-Liver Cancer

Studies have shown that different polar parts of persimmon leaves (ethyl acetate part, n-butanol part and water extraction part) all have tumor-inhibitory effects on mice with mouse hepatoma cell (H22) liver cancer [94]. The compounds of persimmon water extract (PWE) can improve the liver dysfunction caused by lipotoxicity, which is linked to PWE’s ability to regulate oxidative stress, improve mitochondrial dysfunction, and reduce phosphatidylcholine (PCs) and lysophosphatidylcholine (lysoPCs) [95]. Flavonoids isolated from persimmon leaves (PLF) have a strong free-radical-scavenging capacity and can increase the ROS levels in cancer cells (HCT116 (colorectal cancer) and HepG2 (liver cancer cells) and promote apoptosis, indicating that PLF’s anti-proliferative and apoptotic effects on cancer cells are related to oxidative stress [96]. Compared with cyclophosphamide, PLF has fewer side effects and shows anti-cachexia activity. PLF can enhance the immunity of mice and inhibit the growth of liver tumors. The inhibition rate was 49.35% [97]. Synergistic effects with significantly higher tumor-inhibition rates were observed when H22 tumor-bearing mice were treated with a combination of cyclophosphamide and persimmon leaf ethyl acetate (PE). PE was able to enhance the antioxidant capacity of H22 tumor-bearing mice bodies so that the SOD level and pro-apoptotic protein Bax expression in tumor tissues were obviously upregulated, while the MDA level and the expression of the inhibitor apoptotic protein Bcl-2 were downregulated [98]. Of course, in addition to regulating oxidative stress, persimmon leaf extract can also act on other signaling pathways to show therapeutic potential against liver cancer. In cancer cells (HepG2 and Human embryonic kidney 293Acells with high basal JNK (C-Jun *N*-terminal kinase) activity), EEDK leads to JNK-AP-1/p53-mediated cancer cell death by activating the PDGFR-Rac-JNK signaling axis [99]. EEDK can also inhibit hepatocyte growth factor (HGF)-mediated cell migration and invasion, weaken HGF-mediated JNK/C-Jun activation, and reduce HGF receptor Met activity, suggesting that EEDK may treat hepatocellular carcinoma by inhibiting the HGF/Mesenchymal-epithelial transition factor signaling pathway [100].

### 3.4. Prostate Cancer

The total flavonoids extracted from persimmon leaves (FPL) could inhibit the proliferation, migration and induce the apoptosis of human prostate cancer cells (PC-3). By detecting the activities of ROS, MDA, nitrite and inducible nitric oxide synthase (iNOS), it was found that FPL could activate oxidative stress and change mitochondrial membrane permeability, thereby inhibiting cell proliferation, migration and inducing apoptosis and have a certain therapeutic effect on prostate cancer [101]. The anti-prostate cancer activity of flavonoids in persimmon leaves has been supported by few studies [102]. Relevant studies have shown that some flavonoid derivatives from persimmon have significant anti-prostate cancer activities [103,104]. Fisetin has good anti-prostate cancer activity. The treatment of LNCaP (human prostate cancer cells) with fisetin found that fisetin can inhibit LNCaP cells by arresting the cell cycle in the G1 phase, regulating the CKI-cyclin-cdk network, and inducing apoptosis [105]. There are also research findings that fisetin inhibits the Akt signaling pathway, leading to a decrease in the expression of PI3-K (Phosphatidylinositol 3-kinase protein) and the phosphorylation of Thr^308^ and Ser^473^ sites. It also inhibits the growth of prostate cancer cells, promotes apoptosis, inhibits the PI3-K/Akt and JNK signaling pathways, reduces the expression of matrix metalloproteinases (−2 and −9), and inhibits the metastatic ability of PC-3 [106]. In addition to diosquinone, a naphthoquinone epoxide previously isolated from the root bark of Diospyros mespiliformis (Hostch) and D. tricolor [Ebenaceae] has shown anti-prostate cancer activity [107].

### 3.5. Cardio Cerebral Vascular and Myocardial Protection

Many studies have shown that oxidative stress is a key factor in the pathogenesis and subsequent evolution of many diseases. Enhanced oxidative stress on cellular components and alterations in the molecular pathways that support the pathophysiology of cardiovascular disorders are caused by abnormal free radical production. Ang-II promotes the generation of ROS, which leads to the activation of a variety of signaling kinases, which are mostly regulated by ROS. The enhanced expression of procollagen I and III is apparent at the molecular level, as well as significant contractile dysfunction, both of which are closely correlated with increased NADPH-oxidase activation. ROS may damage myofibrillar proteins, causing contractile dysfunction in HF. Changes in ROS levels can also influence the functionality of ion channels and transporters, including calcium channels. P66Shc suppresses the fork head box O (FOXO) transcription factors in the nucleus, resulting in a reduction in the expression of ROS-scavenging enzymes [108]. The prevalence and incidence of cardiovascular disease are currently increasing, so the correlations between oxidative stress and cardiovascular disease had been intensively studied. Persimmon leaves proved to have good activities in cardiovascular diseases, so the mechanism of persimmon leaves in cardio cerebral vascular has been studied.

Ri Ryu found that ethanolic extracts of persimmon (EPL) leaf could prevent and improve thrombosis by inhibiting coagulation and the production of serotonin, thromboxane A2, and soluble P-selectin [42]. Likewise, a mixture of ethanolic extracts of persimmon leaves and Citrus junos Sieb (CJS) can significantly improve coagulation parameters and lipid metabolism disorders in C57BL/6J mice [109]. The regulation of lipid parameters is of great significance for the prevention and treatment of atherosclerosis, but at the same dose, the ability of the phospholipid complexes of total flavonoids from persimmon leaves (PLF-PC) to regulate the levels of total cholesterol (TC), triglycerides (TG), low-density lipoprotein cholesterol (LDL-C), high-density lipoprotein-cholesterol (HDL-C), APOB/APOA1 (apolipoprotein A1, apolipoprotein B) in serum is better than that of PLF, because of the higher bioavailability of PLF-PC [110]. In addition, proanthocyanidins in persimmon leaves can dilate blood vessels through the endothelium-dependent nitric oxide/cGMP pathway and exert antihypertensive effects [111]. EPL shows cardioprotective effects in a rat model of acute myocardial ischemia [112]. Ouyang Ping found that persimmon leaf flavonoids can significantly inhibit the apoptosis of neonatal rat cardiomyocytes, which were induced by hypoxia and reoxygenation and advanced glycation end products [113]. In vitro and in vivo studies have shown that persimmon leaf flavonoids can improve cerebral ischemia tolerance and alleviate cerebral ischemia/reperfusion injury in mice [114].

**Figure 9:**
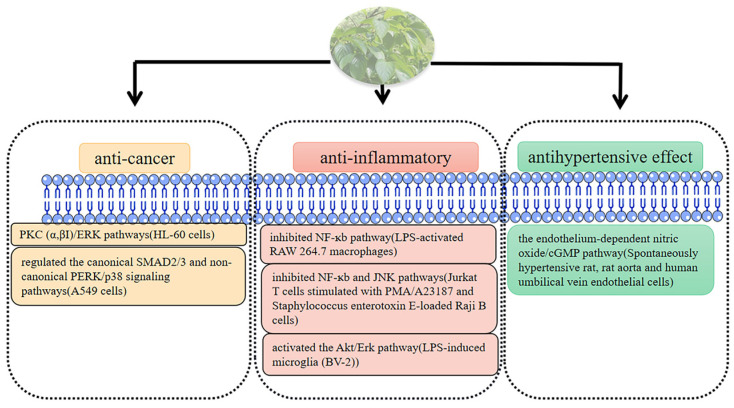
Mechanism of other biological activities in persimmon leave.

Based on the above description, we also summarized the mechanism of other biological activities of persimmon leaves (Figure 9)

## 4. Other Human Diseases

### 4.1. Anti-Lung Cancer

PLF can enhance the cytotoxicity of heavy ion irradiation on lung adenocarcinoma (A549) cells and reduce the phosphorylation of the ataxia telangiectasia-mutated (ATM)-dependent pathway checkpoints during DNA damage, and combination therapy can also reduce tumor volume [115]. Kayoko KAWAKAMI. found that flavonols with the 2″-galloly moiety of PLE can enhance the cytotoxicity of doxorubicin (DOX) to A549 cells and inhibit the phosphorylation of the ATM pathway and protein phosphorylation of related checkpoints in a dose-dependent manner. More significant, however, is that G2/M checkpoints are canceled; the results show that the effect may be related to the presence of gallic acid flavonoid glycosides [116]. Persimmon leaf polysaccharides regulate the canonical Recombinant Mothers Against Decapentaplegic Homolog2/3 and non-canonical phosphorylated extracellular-signal-regulated kinase/p38 signaling pathways and regulate the expression of epithelial marker E-cadherin, mesenchymal markers, *N*-cadherin and vimentin by inhibiting the transforming growth factor-β1(TGF-β1) pathway, thereby inhibiting the EMT (Epithelial-mesenchymal transition) and migration of A549 cells, as well as invasion and anoikis resistance [58].

### 4.2. Acute Promyelocytic Leukemia

Through different pathways of protein kinase C (α, βI)/ERK, an acetone extract of *D. kaki* leaves (KV-1) in combination with low-dose 1,25-dihydroxyvitamin D_3_ [1,25-(OH)_2_D_3_] can induce human promyelocytic leukemia (HL-60) cells to differentiate along the monocyte pathway, whereas stimulation with all-trans retinoic acid (ATRA) induces HL-60 cells to differentiate along the granulocyte pathway, significantly increasing the differentiation level of HL-60 cells. KV-1 not only has the potential to work synergistically with 1,25-(OH)_2_D_3_ or ATRA in the treatment of human promyelocytic leukemia but can also reduce the side effects of both drugs [117].

### 4.3. Anti-Inflammatory

Jung Keun Cho studied the anti-inflammatory effect of PLE and found that PLE could attenuate ultraviolet B (UVB)-induced inflammation in HacaT keratinocytes and mice [118]. Oral administration of PLE resulted in reduced contact dermatitis and ear swelling and decreased lymph node weight in phthalic anhydride (PA)-allergic mice [119]. Supplementation of the diet of ulcerative colitis (UC) models with persimmon-derived tannins significantly reduced disease activity and colonic inflammatory responses, owing to their ability to alter microbiota composition (inhibition of Enterobacteriaceae and Enterococcus expansion) and immune responses, which may make them promising drug candidates for the treatment of chronic inflammatory bowel disease (IBD) [120]. Kyoung-Su Kim isolated two triterpenoids, coussaric acid (CA) and betulinic acid (BA), from persimmon leaves, and their studies showed that CA and BA could inhibit the nuclear factor kappa B pathway in the inflammatory mouse leukemia cells of monocyte macrophage macrophages induced by lipopolysaccharide (LPS), thereby reducing the production of pro-inflammatory cytokines and pro-inflammatory mediators, showing anti-inflammatory potential [121]. In a skin allergy and atopic dermatitis (NC/Nga) mouse model, PLE can alleviate the behavioral response of dermatitis mice, increase serum IgE levels, and significantly inhibit the development of a dermatitis response [122]. Similarly, after oral administration of PLE to Def-sensitized (NC/Nga) mice, the expression of T helper 2(Th2) chemokines (chemokine C-C motif chemokine 17, chemokine C-C motif chemokine 22, chemokine C-C motif chemokine 27) in ear tissue was inhibited, and serum IgE levels were reduced [123]. The anti-inflammatory mechanism of PLE was studied by Hyun-Su Lee, and PLE exhibited inhibitory effects on NF-ĸB and JNK pathways, thereby blocking the activation of T cells in ear tissue and lymph nodes at a non-toxic concentration of 50 μm/mL. PLE can effectively reduce the mRNA level of Interleukin-2 in Junkat T cells, in addition to controlling the infiltration of effector cytokines and mast cells produced by activated T cells [124]. Naoxinqing can regulate the expression of inflammatory factors and activate the Akt/Erk pathway to exert anti-inflammatory and anti-apoptotic effects, which play an important role in the treatment of stroke [125].

## 5. Experimental and Clinical Studies

To evaluate the effect of persimmon leaves, experimental and clinical studies on anti-diabetics, anti-tumor and neuroprotective activity have been carried out, which are summarized in Table 8.

## 6. Conclusions

In recent years, many experimental and clinical studies of traditional Chinese medicine have been conducted, indicating that the pharmacodynamic mechanisms of many natural ingredients are related to oxidative stress. The main compounds of persimmon leaves are flavonoids and triterpenoids, which are potential antioxidants with polyhydroxyl structures. In the follow-up research, it is worth studying the pharmacological activity of triterpenoids with more novel structures. These natural products from persimmon leaves may regulate oxidative stress and treat a variety of diseases. This review has shown that persimmon leaves are important source of antioxidants that can help fight oxidative stress and modulate various pharmacological processes. This provides a new direction for the research on drugs for oxidative stress-related diseases.

## Figures and Tables

**Table 1:** Structure names and numbers of flavonoids in persimmon leaves.

No.	Type	Name	R	References
**1**	A	quercetin	H	[31]
**2**	A	rutin	glc (6→1) rha	[36]
**3**	A	isoquercitrin	glc	[31]
**4**	A	quercetin-3-*O*-β-galactoside(hyperoside)	gal	[31]
**5**	A	quercetin-3-*O*-β-2″-galloylglucoside	2″-galloyl-glc	[31]
**6**	A	quercetin-3-*O*-β-2″-galloylgalactoside	2″-galloyl-gal	[35]
**7**	A	quercetin-3-*O*-β-2″-coumaroylglucoside	2″-coumaroyl-glc	[37]
**8**	B	kampferol	H	[31]
**9**	B	kaempferol-3-*O*-glucoside(astragalin)	glc	[31]
**10**	B	trifolin	gal	[31]
**11**	B	kaempferol-3-*O*-α-l-rhamnopyranoside	rha	[5]
**12**	B	kampferol-3-*O*-β-2″-galloylglucoside	2″-galloyl-glc	[35]
**13**	B	kampferol-3-*O*-β-2″-galloylgalactoside	2″-galloyl-gal	[35]
**14**	B	kaempferol-3-*O*-β-2″-coumaroylgalactoside	2″-coumaroyl-gal	[37]
**15**	B	kaempferol-3-*O*-β-2″-coumaroylglucoside	2″-coumaroyl-glc	[37]
**16**	B	kaempferol-3-*O*-β-2″-feruloylglucoside	2″-feruloyl-glc	[37]
**17**	B	kaempferol-3-*O*-α-arabinoside	ara	[37]
**18**	C	myricetin	H	[32]
**19**	C	annulatin	CH_3_	[38]
**20**	C	myricetin-3-*O*-α-l-rhamnopyranoside	rha	[39]
**21**	C	myricetin-3-*O*-β-d-glucopyranoside	glc	[39]
**22**	C	myricetin-3-*O*-β-d-galactoside	gal	[40]
**23**	D	vitexin	glc	[40]
**24**	D	2″-*O*-rhamnosyl vitexin	glc (2→1) rha	[40]
**25**	D	8-*C*-[α-l-rhamnopyranosyl-(1→4)]-α-d-glucopyranosylapigenin	glc (4→1) rha	[39]
**26**		isorhamnetin		[41]
**27**		isorhamnetin-3-*O*-β-d-glucopyranoside		[36]
**28**		catechin		[42]
**29**		isocatechin		[42]
**30**		epicatechin gallate		[42]
**31**		chrysontemin		[31]

**Table 2:** Structure names and numbers of ursane-type triterpenes in persimmon leaves.

No.	Name	References
**32**	19α-hydroxy ursolic acid	[36]
**33**	α-amyrin	[36]
**34**	24-hydroxyursolic acid	[45]
**35**	uvaol	[36]
**36**	ursolic acid	[36]
**37**	pomolic acid	[46]
**38**	rotungenic acid	[46]
**39**	barbinervic acid	[46]
**40**	pomolic acid methyl ester	[47]
**41**	rosamutin	[48]
**42**	corsolic acid	[46]
**43**	jacoumaric acid methyl ester	[46]
**44**	24-hydroxy ursolic acid	[45]
**45**	kakisaponin A	[49]
**46**	3α, 19α-dihydroxyurs-12-en-24, 28-dioic acid	[46]
**47**	24-hydroxy-3-epi-ursolic acid	[46]
**48**	19, 24-dihydroxyurs-12-en-3-on-28-oic acid	[46]
**49**	rosamultin	[50]
**50**	rotungenicacid-28-*O*-α-l-rhamnopyranosyl-(1→2)-β-d-glucopyranoside	[49]
**51**	28-*O*-α-l-rhamnopyranosyl (1→2)-β-d-glucopyranoside tormentic acid ester	[49]
**52**	2α, 3α, 19α, 24-tetrahydroxyurs-12-en-28-oic acid-28-*O*-β-d-glucopyranosyl ester	[49]
**53**	2α, 3α, 19α, 23-tetrahydroxyurs-12-en-28-oic acid-*O*-β-d-glucopyranosyl ester	[49]
**54**	niga-ichigoside F1	[49]
**55**	kakisaponin C	[50]
**56**	28-*O*-β-d-glucopyranosyl-3α, 24-dihydroxy-19-oxo-18, 19-seco-urs-11, 13 (18)-dien-28-oic acid	[49]
**57**	kakisaponin B	[49]
**58**	28-*O*-β-d-glucopyranosyl-3β, 12β, 19, 24-tetrahydroxy-18, 19-seco-urs-13 (18)-en-28-oic acid	[49]
**59**	28-*O*-β-d-glucopyranosyl-3β, 12β, 24-trihydroxy-19-oxo-18, 19-secours-13 (18)-en-28-oic acid	[49]
**60**	28-*O*-β-d-glucopyranosyl-3β, 12α,19, 24-tetrahydroxy-18, 19-seco-urs-13 (18)-en-28-oic acid	[49]
**61**	3α, 19α-dihydroxyurs-12, 20 (30)-dien-24,28-dioic acid	[46]
**62**	18, 19-seco-3β-hydroxy-urs-12-en-18-one	[51]
**63**	28-*O*-β-d-glucopyranosyl-3α, 24, 30-trihydroxyurs-12, 18-diene-28-oic acid	[49]

**Table 3:** Names and numbers of other types of triterpenoid structures in persimmon leaves.

No.	Name	References
**64**	oleanolic acid	[46]
**65**	β-amyrin	[48]
**66**	spathodic acid	[52]
**67**	24-hydroxy-3-epi-oleanolic acid	[52]
**68**	maslinic acid methyl ester	[47]
**69**	3R, 24, 29-trihydroxyolean-12-en-28-oic acid	[53]
**70**	3α, 24, 29-trihydroxyolean-12(13)-en-28-oic acid-*O*-β-d-glucopyranoside	[49]
**71**	ryobunin C	[49]
**72**	2α, 3α, 19α, 24 tetrahydroxyolea-12-en-28-oic acid-β-d-glucopyranosyl ester	[49]
**73**	28-*O*-β-d-glucopyranosyl-3β, 1 2β, 19, 24-tetrahydroxy-18, 19-seco-ole-13 (18)-en-28-oic acid	[49]
**74**	lupeol	[48]
**75**	betulinic acid	[46]
**76**	betulin	[54]
**77**	kakidiol	[50]
**78**	friedelin	[54]
**79**	glutinol	[54]

**Table 4:** Structure names and numbers of monoterpenes, sesquiterpenes and diterpenes in persimmon leaves.

No.	Name	References
**80**	Linarionoside A	[48]
**81**	Linarionoside B	[48]
**82**	blumeol C glucoside	[48]
**83**	byzantionoside B	[48]
**84**	vomifoliol 9-*O*-α-arabinofuranosyl (1→6)-β-d-glucopyranoside	[57]
**85**	persimmonoid A	[55]
**86**	persimmonoid B	[55]
**87**	ligustroside	[55]
**88**	oleuropein	[55]
**89**	1β-hydroxy-4 (15), 5E, 10 (14)-germacratriene	[61]
**90**	teucdiol A	[61]
**91**	selin-4 (15)-en-1β, 11-diol	[61]
**92**	Abbeokutone	[54]
**93**	trihydroxykaurine 3α, 6α, 17-trihydorxykaurane	[54]

**Table 5:** Structure names and numbers of phenylpropanoids, steroids and alkaloids in persimmon leaves.

No.	Name	References
**94**	(+)-medioresinol	[55]
**95**	(+)-syringaresinol	[55]
**96**	(+)-pinoresinol	[55]
**97**	(+)-medioresinol monoglucoside	[55]
**98**	(+)-syringaresinol-β-d-glucoside	[55]
**99**	(+)-pinoresinol-β-d-glucoside	[55]
**100**	(−)(7′*S*, 8*S*, 8′*R*)-4,4′-dihydroxy-3, 3′, 5, 5′-tetramethoxy-7′, 9-epoxylignan-9′-ol-7-one	[55]
**101**	(+)-isolariiresinol	[55]
**102**	4, 4-dihydroxy intercoca acid	[55]
**103**	diospyrin	[62]
**104**	diosprol	[62]
**105**	6-hydroxy-7-methoxycoumarin	[53]
**106**	scopolamine (6-methoxy-7-hydroxycoumarin)	[53]
**107**	daucosterol	[63]
**108**	β-sitosterol	[63]
**109**	tatarine C	[38]

**Table 6:** Structure names and numbers of phenolic acids in persimmon leaves.

No.	Name	References
**110**	gallic acid	[64]
**111**	kakispyrone	[38]
**112**	kakispyrol	[65]
**113**	protocatechuic acid	[46]
**114**	benzoic acid	[46]
**115**	*p*-hydroxybenzoic acid	[46]
**116**	salicylic acid	[53]
**117**	furoic acid	[53]
**118**	syringic acid	[53]
**119**	vanillic acid	[53]
**120**	hydroxytyrosol	[56]
**121**	*C*-veratroylglycol	[56]
**122**	3-(4-hydroxyl-3-methoxyphenyl) propane-1, 2-diol	[56]
**123**	methyl coumarate	[56]
**124**	4-allyl pyrocatechol	[56]

**Table 7:** Effect of persimmon leaf extract complex or combination of persimmon leaf and other drugs in treating diseases.

Medicine	PLF-PC	CTX	CTX	DOX	Heavy Ion Radiotherapy
Persimmon leaf	+	+(PLF)	+(PE)	+(PLE)	+(PLF)
Diseases	atherosclerosis	liver cancer (H22)	liver cancer (H22)	lung cancer (A549)	lung cancer (A549)
Effect	1. Improve oral bioavailability	1. Reduce side effects 2. Develop immunity from disease	1. Regulation of oxidative stress 2. Increased tumor suppression rate	1. Increased toxicity to cancer cells	1. Increased toxicity to cancer cells

**Table 8:** Experimental and clinical studies regarding the use of persimmon leaves.

Main Objective	Conclusion	References
	Anti-diabetics	
To investigate the effects of different solvent extracts from persimmon leaves on the antioxidant capacity of streptozotocin (STZ) diabetic model mice	Improving the antioxidant capacity of diabetic mice may be one of the mechanisms of the hypoglycemic effect of ethyl acetate extract and alcohol precipitation extract from persimmon leaf leaves	[12]
To evaluate the hypoglycemic effect of aqueous extract of persimmon leaves on a mouse model of diabetes	Persimmon leaf extract exhibits considerable anti-diabetic effects by inhibiting α-glucosidase and maintaining the function of β-cells	[78]
To study the efficacy of persimmon leaf extract in patients with prediabetes	Based on proteomic changes in different body fluids obtained by prediabetic patients after controlling PLE intake, it has been shown that persimmon leaf extract can improve blood sugar levels	[26]
To study the effects of persimmon leaf supplementation on mice with type 2 diabetes	Persimmon leaves ameliorate hyperglycemia by altering the activity and mRNA expression of liver enzymes involved in glucose utilization and glucose production, and also ameliorate dyslipidemia and hepatic steatosis by combining a decrease in hepatic lipogenesis and an increase in fecal fat excretion	[77]
	Anti-tumor	
The crude polysaccharides in persimmon leaves were used as the research objects, and their anti-tumor and anti-metastatic activities were evaluated by oral administration in mice.	Crude polysaccharides in persimmon leaves induce natural killer (NK) cells-mediated tumoricidal activity and inhibit tumor metastasis in mice in a dose-dependent manner.	[77]
The purpose of this study was to investigate the anti-cancer properties of flavonoids isolated from persimmon leaves.	Flavonoids isolated from persimmon leaves (PLF) can induce apoptosis of HCT116 (colorectal cancer) and HepG2 (liver cancer) cells, and the intracellular ROS level is increased. In addition, PLF has a strong ability to scavenge free radicals. The anti-proliferative activity of PLF on cancer cells is related to the induction of apoptosis and oxidative stress.	[96]
This study investigated the effect of persimmon leaf extract on cellular DNA damage checkpoint signaling on cancer chemotherapy sensitivity.	Persimmon leaf extract inhibits ATM activity during DNA damage response in A549 lung adenocarcinoma cells induced by doxorubicin.	[97]
To study the anti-tumor and immunomodulatory activities of total flavonoids extract from persimmon leaves on H22 hepatoma mice.	The total flavonoids extract of persimmon leaf can effectively inhibit the growth of liver tumors in vivo by enhancing the immune function of mice, showing the potential of a safe and effective anti-cancer drug or functional immune enhancer.	[117]
	Neuroprotective activity	
The effects of ethanol extract of flavonoid-rich persimmon leaf on APP/PS1 transgenic mice were studied by oral administration.	Alleviate cognitive deficits, amyloid production, oxidative stress, and neuroinflammation in APP/PS1 transgenic mice.	[98]
The protective effects and mechanisms of flavonoid-rich ethanol extracts on the cortex and hippocampus of D-galactose aged mice were studied.	Flavonoid-rich ethanol extract of persimmon leaf attenuates D-galactose-induced oxidative stress and neuroinflammation-mediated brain aging in mice.	[92]
APP/PS1 mice were used as AD models to investigate whether the protective effect of flavonoids extracted from persimmon leaves on the synapses of AD mice was related to Rho GTPases activity.	It significantly inhibited RhoA-GTP activity, improved learning and memory function, and antagonized the downregulated expression of synaptophysin and synapse-associated proteins.	[91]
To investigate the neuroprotective effect of persimmon leaf flavonoid extracts in an in vivo model of focal ischemia/reperfusion (I/R) injury induced by middle cerebral artery occlusion (MCAO) and transient global cerebral ischemia (4-VO) due to quadruple vascular occlusion.	Significantly protects rats from MCAO and 4-VO ischemic injury, protects hippocampal neurons from glutamate-induced excitotoxic damage, and protects cortical neurons from hypoxia-induced damage in vivo. Useful in the prevention and treatment of related neurodegenerative diseases such as ischemia/reperfusion injury.	[89]

## Data Availability

Not applicable.
